# Robot occupations affect the categorization border between human and robot faces

**DOI:** 10.1038/s41598-023-46425-0

**Published:** 2023-11-07

**Authors:** Junyi Shen, Guyue Tang, Shinichi Koyama

**Affiliations:** 1 University of Tsukuba, Graduate School of Comprehensive Human Sciences, Tsukuba, 305-8574 Japan; 2 University of Tsukuba, Institute of Art and Design, Tsukuba, 305-8574 Japan

**Keywords:** Human behaviour, Cognitive neuroscience, Social neuroscience

## Abstract

The Uncanny Valley hypothesis implies that people perceive a subjective border between human and robot faces. The robot–human border refers to the level of human-like features that distinguishes humans from robots. However, whether people’s perceived anthropomorphism and robot–human borders are consistent across different robot occupations remains to be explored. This study examined the robot–human border by analyzing the human photo proportion represented by the point of subjective equality in three image classification tasks. Stimulus images were generated by morphing a robot face photo and one each of four human photos in systematically changed proportions. Participants classified these morphed images in three different robot occupational conditions to explore the effect of changing robot jobs on the robot–human border. The results indicated that robot occupation and participant age and gender influenced people’s perceived anthropomorphism of robots. These can be explained by the implicit link between robot job and appearance, especially in a stereotyped context. The study suggests that giving an expected appearance to a robot may reproduce and strengthen a stereotype that associates a certain appearance with a certain job.

## Introduction

In recent years, the development of robotics and artificial intelligence technologies has led to an increasing number of robots fulfilling different occupational roles, such as industrial, social, service, security, and culinary^1^. The current robotics industry considers implementing anthropomorphic features to robots as essential. Giving robots anthropomorphic features can increase users’ perceived anthropomorphism, trust, and favorability toward robots^2–4^. Therefore, to attract customers, humanoid robots have become increasingly in demand in various jobs. Consequently, making robots look like humans has also become popular^5^. However, anthropomorphic designs for robots are not always favored, and the anthropomorphism of robots remains a contentious topic^6^.

### Robot–human border and perceived anthropomorphism

The threshold of human-like appearance that differentiates humans from robots is termed the robot–human border. The human resemblance that helps increase perceived anthropomorphism can come either from a robot’s appearance or from other aspects including the robot’s language, movements, and functions^6,7^. A robot’s physical appearance is a cardinal factor in perceptual anthropomorphism^8^. As the proportion of human appearance a robot possesses increases, the degree of anthropomorphism people perceive in the robot also increases. When a robot reaches a certain level of anthropomorphism, people will begin to recognize the robot as human. The proportion of human image that distinguishes humans from robots is referred to as the robot–human border.

The most widely known theory on this is the “Uncanny Valley.” It suggests that as the similarity of a robot to humans increases, its relatability also increases. However, a robot that is very similar to but not identical with a human will risk eliciting strong negative emotions in users. Once a robot is indistinguishable from a human, it will be welcomed like a human^9^. However, Robertson^10^ suggested that the Uncanny Valley is related to the stereotype that a robot should look a certain way. Viewers feel uncomfortable when robots have an appearance that does not fit this stereotype. If people expect a robot to have a low-level anthropomorphic appearance, they will feel uncomfortable seeing instead a robot with a high-level anthropomorphic appearance. People have different expectations of humans and robots for different jobs^11^. If the Uncanny Valley is influenced by our expectations of a robot’s appearance, then the border between humans and robots may change based on the robot’s occupation.

As people’s perceived anthropomorphism of a robot increases gradually, the robot gets closer to the robot–human border, until it reaches the point of crossing it. According to existing studies, people’s perception of robot anthropomorphism is influenced by various factors, including the robot’s attributes (robot appearance and behavior) and the observer’s characteristics (motivation, social context, age, and gender)^12–16^. Under different observation conditions, people may perceive different degrees of anthropomorphism for robots with the same appearance. The border between robots and humans may be influenced by factors associated with social attributes. People have different preferences regarding the anthropomorphism of robots in different domains^17^. The robot’s occupation may be a potential factor influencing the robot–human border. The next section explores the influence of occupation on people’s perceived robot anthropomorphism.

### Robot occupation and the robot–human border

As an essential social classification, various stereotypes accompany different occupations^18^. Service personnel are expected to have high warmth attributes, whereas security guards are perceived as protecting only the wealth of the rich and promoting the gap between the rich and the poor^19,20^. Occupational classifications influence our perceptions and expectations of others in different directions and degrees^21^. With the development of robotics and the widespread use of various robots, people have developed stereotypes about robots engaged in different occupations. They hold different preferences around the anthropomorphism, personality, ability, and expectations of robots engaged in different occupations^22,23^. For example, consumers are more likely to prefer a warm robot for travel services and an intelligent one for financial services^24^.

A robot’s occupation may influence people’s anthropomorphic preferences. Consumers make higher predictions of the quality of food produced by highly anthropomorphic robot chefs than by less anthropomorphic ones^25^. In the industrial and social domains, people prefer a low and high degree of anthropomorphism, respectively; whereas in the service domain, there is no clear preference^17^. The positive effect of the anthropomorphic features of robots may depend on the context in which they are used^26,27^. People tend to favor robots in occupations where its human likeness matches the sociability expected in such positions^28^.

Different occupations influence people’s preferences for anthropomorphism, and higher expectations may lead to higher satisfaction standards^29,30^. This study hypothesizes that when people have higher anthropomorphism expectations for robots engaged in a specific occupation, it would be more challenging to perceive precise anthropomorphism to recognize them as human. Robots in occupations with high expectations of anthropomorphism would need to have more human-like features to cross the border from robot to human. The human similarity represented by the robot–human border will vary by occupation. Thus, the following hypotheses were proposed:

H1: Robot occupation influences the human photo proportion at the perceived robot–human border.

Service and security robots are widely used and studied^31,32^. Service personnel and security guards have different societal stereotypes^19,20^. This study selected robots, service robots, and security robots as three occupational categories and investigated the effect of robot occupation (robot vs. service robot vs. security robot) on human photo proportion represented by the robot–human border using a classification task of morphed face images.

H2: Participant age influences the human photo proportion at the perceived robot–human border.

People’s perceptions of anthropomorphism and preferences for robots may differ by age. Older people prefer anthropomorphic robot designs^33^ and a purely mechanical or human appearance as opposed to younger people who are more receptive to a hybrid appearance^34^.

H3: Participant gender influences the human photo proportion at the perceived robot–human border.

Whether users of different genders perceive the anthropomorphism of robots differently remains to be explored. A study of robots for children found that girls preferred humanoid robots more than boys did^35^. In another study, males (compared to females) experienced greater pleasure from interacting with highly anthropomorphic robots. Thus, gender may influence the robot–human border.

### Point of subjective equality (PSE)

While observing visual stimuli, do people recognize an object with human-like features as human? Answering this has important implications for locating the border between robots and humans. In this study, the point of subjective equality (PSE) was defined as the human photo proportion of the morphed face image at which a participant is equally likely to classify the image as a robot or human. It represents the subjective midpoint between the robot and human anchor values the participant perceived in classification tasks. Researchers have often manipulated the human photo proportion of stimuli in a stepwise manner and recorded participants’ responses to and categorization of stimuli. Some studies treat the human photo proportion of stimuli as human similarity, although they are not equivalent^36–38^. Using the concept of the human photo proportion rather than human similarity can more accurately match the results of the experiments. Research has not been able to show the value of the human photo proportion of stimuli that is reached when observers begin identifying stimuli as more human than robot^7^. Therefore, this study used a psychophysical method to measure the robot–human border. Participants performed a human or robot classification task on images containing a controlled proportion of human faces. This task helped estimate the PSE. The experiment can support the search for the degree of human photo proportion as represented by the robot–human border. This study aimed to investigate the influence of robot occupation (robot vs. service robot vs. security robot) on the human similarity represented by the robot–human border using a classification task of morphed face images. In addition, it examined the effects of participants’ gender and age on perceived robot anthropomorphism.

## Methods

This study examined the impact of robot occupations on users in relation to the robot–human border based on occupational classification. Three robot occupations, namely robot, service robot, and security robot, were selected. Corresponding to the three robot occupations, three classification tasks were conducted on morphed face images to examine the impact of robot occupations on the robot–human border. Participants’ age and gender were examined as potential influences.

### Participants

The experiments involving human participants were reviewed and approved by the Research Ethics Committee of the Art and Design Faculty of the University of Tsukuba, No. G22-12. All methods were performed in accordance with the Declaration of Helsinki. Informed consent was obtained from all participants prior to data collection.

A total of 1159 participants from Japan were recruited in January 2023 through Yahoo! Crowdsourcing, a leading online survey platform in Japan. Participants received points that could be used instead of money at certain stores in recognition of their contribution. The smallest effect size of interest of $\eta ^2$ = 0.03 was used in sample size planning^39^. Based on an a priori power analysis using G*Power with a power (1 − $\beta$) set to 0. 95 and $\alpha$ = 0.05, the targeted sample size was 600.

A verification code and screening question were used to exclude incomplete responses as the evaluation criteria. The screening question was: “Which of the following is not mentioned in this survey: (i) service robot, (ii) medical robot, (iii) security robot.” Those who chose option (i) or (iii) were excluded. Of the 1159 participants, 1024 completed the survey and answered the screening question and verification code correctly. The group aged between 30 and 39 years included 259 males (*M* = 35.59, *SD* = 2.72) and 251 females (*M* = 35.26, *SD* = 2.94). The group aged between 60 and 69 years included 286 males (*M* = 63.70, *SD* = 2.88) and 228 females (*M* = 63.36, *SD* = 2.84). Participants were also asked about their profession. The sample included people with backgrounds in robot-related (n = 3), service (n = 16), security (n = 17), and other (n= 988) domains.

### Stimuli

**Figure 1:**
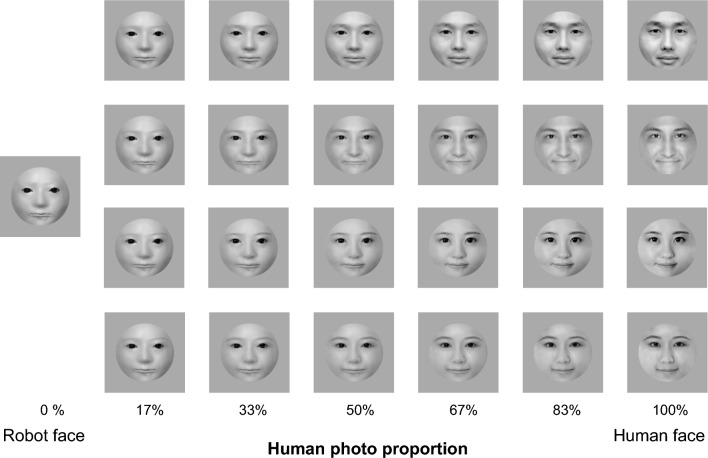
The stimuli used in the experiment.

A total of 25 images (Fig. 1) were used. Face pictures of one robot, two males, and two females were selected. The robot face was sourced from the Telenoid, an android with a minimal human design comprising only the minimum human appearance^40^. Two photos each of male and female human faces were selected from the free material available on the Pakutasso database^41^.

After the human and robot face images were selected, the hair was removed from the pictures, and all five images were adjusted to the round shape to hide the actual face shapes. To avoid the effect of skin color, the images were converted into grayscale pictures with the same luminance in Photoshop. The free online morph tool Webmorph^42^ was used to create the 7-level equally stepped morphed images with human photo proportion from 0 to 100%. The image with 0% human photograph proportion was the original robot face picture, and the image with 100% human photo proportion was an original human face image. Each image was placed in the center of a 710 px * 710 px square gray background image. The luminance of the gray background on the MacBook Pro display was 29.61 cd/m².

### Procedure

The questionnaire was circulated through SurveyMonkey and the participants were recruited using an online survey platform, Yahoo! Crowdsourcing. Each participant was able to use a link to complete the online survey by following the instructions. Only the participants who used desktops were allowed to participate. They were informed about the general terms and conditions. At the beginning of the experiment, they were asked to ensure that they had normal or corrected-to-normal visual acuity. Participants were asked to keep the test environment quiet and bright and to not use any other multimedia devices such as music and TV during the experiment. The display size was suggested to be 10.5 inches or more to present the images. We asked the participants to regulate the brightness of the screen so that it was not harsh on their eyes. Each participant was asked to adjust their seat position to ensure that their face was about 60 cm from the screen. After confirming these preparations, the online survey began.

The questionnaire was developed to explore participants’ identification discrepancy of morphed faces with different robot occupational contexts. The survey had three blocks corresponding to three types of robot occupations. The following pairs of nouns were used as multiple-choice options: “human/robot,” “human/service robot,” and “human/security robot.” Each of the 25 images was projected on separate pages of the questionnaire. Below the image was a two-choice question: “Which of the following options do you think this image looks like”? The options comprised a random pair of the three selected noun pairs, such as “human” and “service robot.” Participants were asked to determine whether the picture was that of a human or of a random type of robot from among the three options, namely robot, service robot, and security robot. There was no time limit. Participants completed the three blocks one by one. The orders of the three blocks and 25 images within each block were randomized.

After completing 75 categorization tasks, participants answered questions about their personal information, including gender, age, and occupation. At the end of the questionnaire, there was one screening question to select the people who had read the question carefully. Only those who answered the screening question and verification code correctly received a reward. The results were statistically analyzed using IBM SPSS Statistics 29.0.

## Results

**Table 1:** Results of a three-way analysis of PSE (robot occupation, participant age, and participant gender), *p < 0.05; **p < 0.01.

Effects	SS	*df*	MS	*F*	*p*	$\eta$
Robot occupation	0.135	2	0.067	4.390	0.012*	0.055
Participant age	1.250	1	1.250	81.521	<0.001**	0.161
Participant gender	0.628	1	0.628	40.966	<0.001**	0.114
Robot occupation × participant age	0.004	2	0.002	0.119	0.888	<0.001
Robot occupation × participant gender	0.001	2	0.000	0.021	0.979	<0.001
Participant age × participant gender	0.134	1	0.134	8.746	0.003**	0.055
Robot occupation × participant age × participant gender	0.012	2	0.006	0.384	0.681	<0.001
Error	46.911	3060	0.015			

There were significant main effects for the three factors but a significant interaction occurred only between participant age and gender.

**Table 2:** Effects of robot occupation, participant age and participant gender on PSE.

Effects	Statistical significance	Compare the PSE value	Hypothesis results
A. Robot occupation	Significant	Service robot (M = 0.56, SD = 0.127) > robot (M = 0.545, SD = 0.123); Service robot (M = 0.56, SD = 0.127) > security robot (M = 0.547, SD = 0.129)	Support H1
B. Participant age	Significant	60s (M = 0.570, SD = 0.132) > 30s (M = 0.531, SD = 0.117)	Support H2
C. Participant gender	Significant	Female (M = 0.565, SD = 0.126) > male (M = 0.538, SD = 0.125)	Support H3
B × C			
At the 30s	Significant	30s × female (M = 0.539, SD = 0.114) > 30s × males (M = 0.524, SD = 0.119)	Support H2
At the 60s	Significant	60s × female (M = 0.593, SD = 0.132) > 60s × male (M = 0.551, SD = 0.130)	Support H2
At male	Significant	Male × 30s (M = 0.524, SD = 0.119) < males × 60s (M = 0.551, SD = 0.130)	Support H3
At female	Significant	Female × 30s (M = 0.539, SD = 0.114) < female × 60s (M = 0.593, SD = 0.132)	Support H3

A three-way analysis of variance (ANOVA) was performed to determine the effects of robot occupation (robots, security robots, and service robots), participant gender (female and male), and participant age (those in their 30s and those in their 60s) on PSE results using the factor composites. Table 1 shows the results. The data were checked and it was confirmed that the error variance of the dependent variables was equal across the groups. The results showed that each group of data conformed to the normal distribution (*p* > 0.05). Based on Mauchly’s test of sphericity, the error covariance matrix of the orthonormalized transformed dependent variables was proportional to an identity matrix (*p* > 0.05). When statistical differences were observed, post hoc tests with Bonferroni correction for multiple comparisons were conducted. In the three-way ANOVA, all the main effects were significant. Only the interaction between participant age and participant gender was significant.

### Point of subjective equality (PSE)

**Figure 2:**
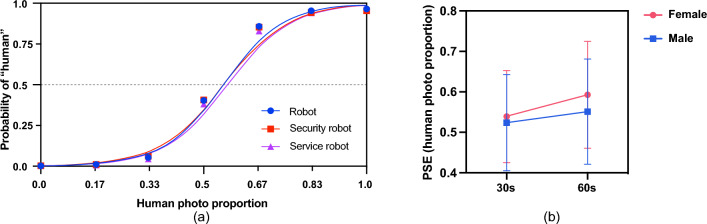
(**a**) Mean probability of ‘human’ responses as a function of the image for all the participants. (**b**) Mean ratings of the average point of equality (PSE) for different age (the 30s vs. the 60s) and gender (female vs. male) groups. Error bars denote standard errors of the mean.

The classification data obtained in the image distinguishing processes may be quantified as the proportion of human responses the participant made at each image for each robot occupation. We marked the “human” answer as “1” and the other answers as “0” in the distinguishing tasks. We scored the morphed images according to the human photo proportion, respectively, including 0, 0.17, 0.5,0.67, 0.83, and 1. Then, we input the answer and human photo proportion together to calculate the PSE for each task using R Studio. As seen in Fig. 2a, the PSE for each task was the value of the human photo proportion at which the probability of ‘human’ was 0.5. The PSE value indicates the human photo proportion of the image at which a participant is equally likely to classify the image as a human or one type of robot. It represents the subjective midpoint between the robot and human anchor values that the participant perceived in the experiment. An increase in the PSE (a rightward shift of the curve) shows that the participants chose ‘human’ more often; inversely, a decrease in the PSE (a leftward shift of the curve) shows that the participants were biased toward choosing “robot/service robot/security robot”.

### Participant age and gender

As shown in Fig. 2b, there was a significant interaction between participant age and gender on PSE, *F* (1, 3060) = 8.746, *p* = 0.003, and $\eta$ = 0.055. No other two-way or three-way interactions were significant. Post-hoc mean comparisons with Bonferroni correction indicated statistically significant mean differences between the PSE of females who were in their 30s versus males who were in their 30s (mean difference (30s female–30s male) = 0.015, *p* = 0.015, 95%CI = [0.003, 0.028]). The PSE of females who were in their 60s was significantly higher than that of males who were in their 60s (mean difference (60s female–60s male) = 0.042, *p* < 0.001, 95%CI = [0.030, 0.054]).

For females, we observed significant differences between the PSE of those in their 30s and 60s, mean difference (30s female–60s female) = − 0.054, *p* < 0.001, 95%CI = [− 0.067, − 0.041]. Similarly, for males, we observed significant differences between the PSE of those in their 30s and 60s, mean difference (30s male-60s male) = − 0.027, *p* < 0.001, 95%CI = [− 0.039, − 0.015]. Table 2 presents a detailed description of the above results.

### Robot occupation

**Figure 3:**
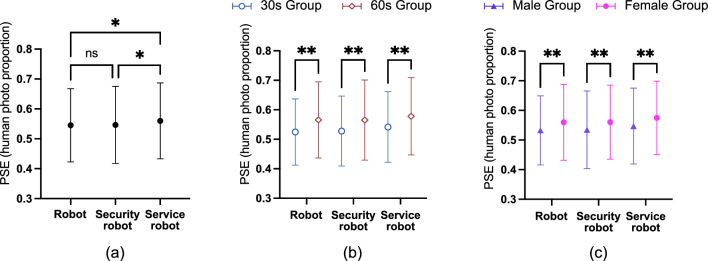
(**a**) Point of subjective equality (PSE) is indicated for the robot, service robot, and security robot. (**b**) The average point of subjective equality (PSE) of the service robot, security robot, and robot for the groups in their 30s and 60s. (**c**) The average point of subjective equality (PSE) of the service robot, security robot, and robot for females and males. Error bars denote standard errors of the mean. *p < 0.05, **p < 0.01.

There was a significant main effect of robot occupation. As shown in Fig. 3a, the PSE of the service robot revealed a significant difference compared to the robot, *F*(2, 3060) = 4.39, *p* = 0.012, and $\eta$ = 0.055. Post-hoc mean comparisons with Bonferroni correction indicated that the PSE of service robots was significantly higher than those for robots (mean difference (service robot-robot) = 0.015, SE = 0.005, *p* = 0.024, 95%CI = [0.001, 0.028]) and security robots (mean difference (service robot-security robot) = 0.014, SE = 0.005, *p* = 0.041, 95%CI = [0.000, 0.027]). The PSE showed whether the participants presented differences in their judgment of anthropomorphism toward either an underestimation or overestimation of various occupational robots.

### Participant age

Participant age had a significant main effect on PSE, *F*(1, 3060) = 81.521, *p* < 0.001, and $\eta$ = 0.161. Table 1 shows that robot occupation and participant age did not have significant interaction effects on PSE, *F*(2, 3060) = 0.119, *p* = 0.888, and $\eta$ < 0.001. Figure 3b illustrates that the two age groups differed for PSE. The mean PSE for those in their 60s was significantly higher than that for those in their 30s, with a mean difference (60s-30s) = 0.040, SE = 0.004, *p* < 0.001, 95%CI = [0.032, 0.049].

### Participant gender

Participant gender had a significant main effect on PSE, *F*(1, 3060) = 40.966, *p* < 0.001, and $\eta$ = 0.114. Table 1 shows that robot occupation and participant gender did not have significant interaction effects on PSE, *F*(2, 3060) = 0.021, *p* = 0.979, and $\eta$ < 0.001. Figure 3c shows that the two gender groups differed for PSE. The mean PSE result was more prominent for females than for males, with the mean difference (female–male) = 0.029, SE = 0.004, *p* < 0.001, 95%CI = [0.020, 0.037].

Based on the results of the three-way ANOVA, we observed that people showed different PSEs of human photo proportion toward morphed images with different occupation contexts. The morphed face image needed to have a higher human photo proportion to be judged as human in the “human/service robot” task, when compared to the “human/robot” and “human/security robot” tasks. Participants showed significant differences in terms of age and gender. There was a significant interaction effect between participant age and gender. Table 2 presents the detailed description and comprehensive summary of the above experimental results and shows the effects of robot occupation, participant age, and participant gender on PSE. Therefore, H1, H2, and H3 were supported.

## Discussion

This study investigated the effects of robot occupation and participants’ gender and age on human photo proportion as suggested by the perceived robot–human border. We analyzed the results of the classification of morphed face images with different levels of human photo proportion using the PSE measurement.

The results primarily support the H1 that robot occupation influences the human photo proportion at the perceived robot–human border. As expected in H1, compared to robots and security robots, participants had the highest human photo proportion for the service robot–human border. This result is consistent with studies that indicate that people have different expectations around the anthropomorphism of robots engaged in different occupations^22^. Participants anticipate greater degrees of anthropomorphism in professions involving more direct human interactions such as education and hospitality^43^. Service robots, compared to security robots and robots, have more direct interactions with humans. Moreover, a prior study revealed that people desire service robots to cater to and recognize the emotional needs of both customers and service providers. This underscores a pronounced demand for the humanization of service robots, which is intrinsically linked to the service industry’s inherent nature^44^. From this, we deduce that participants likely have higher humanization expectations for service robots in contrast to security robots, resulting in elevated PSE outcomes.

People have different anthropomorphic preferences while selecting robots from different occupations^24^. Although PSE was significantly higher for service robots than for robots and security robots, there was no significant difference in human photo proportion as suggested by the robot–human border for robots and security robots. People expect service robots to look more like humans than security robots. This indicates the diversity of influences on the robot–human borderline caused by robot occupations. The results may support Robertson’s view that people’s expectation regarding the appearance of humans in a particular job is reproduced in the formation of robots in a particular job, which could further strengthen their expectation of the appearance of others in a particular job^10^.

This study morphed the images of both male and female humans in order to reduce the additional effect of gender stereotypes for different occupations. As the skin color of the robot has a significant impact on the outcome of human perception^45^, grayscale pictures were used to reduce potential influences on human similarity. Thus, compared to the experimental material that uses robot pictures directly, the experiments in this study controlled for more potential variables to enhance the reliability of the results.

The results supported H2: participant age influences the human photo proportion at the perceived robot–human border. Older adults showed higher PSE scores than younger adults. Compared to younger adults, older adults perceived less anthropomorphism in the same morphed face images. Studies show that older adults prefer robots with high anthropomorphism compared to younger adults^33^. This study hypothesized that as people’s preferences and expectations for human photo proportions of robots increase, morphed face images will have higher human photo proportions to meet the robot–human borderline, resulting in a lower perceived anthropomorphism of the image. The reason for this age difference remains uncertain. It may perhaps be the result of the differences in educational background and socio-cultural factors that cause differences in the robot–human border. This finding serves as an essential reference for the design of robots targeting older adults. Although older adults have stronger negative attitudes toward robots than do younger adults^46^, adopting a robot design that meets the anthropomorphism expectations of older adults may improve their attitudes toward and acceptance of robots. A study revealed that three-year-old children tend to ascribe biological properties to robots more frequently than older individuals^47^. Robots with a high degree of anthropomorphism can be perceived as having attributes akin to a “mind” or “consciousness,” resonating with the tenets of Animism. Such perceptions may shape human-robot interactions, rendering them more intuitive and deeply engaging.

Significant gender differences in PSE results were observed among participants, which supports H3: participant gender influences the human photo proportion at the perceived robot–human border. Females showed higher PSE than males. This result highlights the need for robots to have more human features in order to be recognized as human by females (when compared to males). Thus, it can be said that females (compared to males) have higher expectations and preferences for the proportion of humans in a robot’s face appearance, leading to higher PSE values. This study fills a research gap by examining the effect of gender on the perceived anthropomorphism of robot faces. Studies have found higher perceived anthropomorphism in robot movements among females (when compared to males)^48^. While this study supports the idea that females (when compared to males) show lower perceived anthropomorphism for robots’ faces, this inconsistency may suggest that static robot appearance and dynamic robot movements may lead to different gender effects in perceived anthropomorphism.

There was a significant interaction effect between participant age and gender on PSE. Some cultural and physiological factors might cause such a specific interaction. Although in both age groups, the PSE scores of females were significantly higher than those of males, the gender difference in PSE for those in their 60s was more pronounced than for those in their 30s. In the field of anthropomorphic robot research, we seldom find an interaction between age and gender. This result suggests the importance of controlling for the age and gender of participants in studies of robot anthropomorphism.

## Limitations

Although we attempted to understand the experimental results in relation to occupational stereotypes, each person may have different impressions and expectations of robots in various occupations^49^. There are multidimensional differences in occupational stereotypes of service personnel and security guards in human societies^19,20^; therefore, it is difficult to determine the differences in occupational stereotypes that lead to differences in expectations of anthropomorphism. It is uncertain whether the stereotypes of occupation in human society are consistent with those of robots with the same occupation. Since people’s preferred robot appearance does not necessarily match occupational stereotypes, we cannot simply assume that anthropomorphic preferences for robots vary by occupation. Thus, it remains unclear how the stereotypical views of robots accurately affect human society. To answer this, an in-depth classification and quantitative analysis of specific occupational stereotypes must be conducted in the future. There is a need to systematically investigate the effects of occupation on other dimensions of robot anthropomorphism. Furthermore, understanding how context influences professional stereotypes and preferences for anthropomorphic robots is a pressing question that demands an answer. Anthropomorphism was achieved in this study by presenting the participants with pictures of anthropomorphic faces; however, the anthropomorphism of robots can transcend mere appearance to include communication, context, or range of behavioral variability^50,51^. Occupation, as a classification method for social roles, is employed as the primary categorization approach in this study. However, other types of social role classifications might also have potential significance in future robot research, such as the robot’s skin color, gender, and power position. Moreover, the interaction effect between participant age and gender on PSE warrants deeper exploration.

## Conclusion

This study found that robot occupations influence the perceived robot–human border. Compared to robots and security robots, service robots require more human photo proportions in order to be recognized as humans. Older adults chose images with higher human photo proportion to be human, as suggested by the robot–human border in all three occupational contexts, when compared to younger adults. Females showed higher PSE than did males. People have different anthropomorphism expectations for robots in various occupations, as seen in the human photo proportion suggested by the robot–human border. Robots should be studied carefully and designed to suit diverse occupations appropriately. This study highlighted the importance of focusing on robot occupation and participants’ age and gender and serves as an essential reference for research on robot anthropomorphism.

## Data Availability

The raw data are available upon request to the corresponding author.
